# Low sero-prevalence of hepatitis delta antibodies in HIV/ hepatitis B co-infected patients attending an urban HIV clinic in Uganda

**DOI:** 10.4314/ahs.v17i4.4

**Published:** 2017-12

**Authors:** Elizabeth Katwesigye, Emmanuel Seremba, Fred Semitala, Ponsiano Ocama

**Affiliations:** 1 Department of Medicine, Mulago Hospital and Makerere University College of Health Sciences, Kampala, Uganda; 2 Department of Medicine, Mulago Hospital, Kampala, Uganda

**Keywords:** Co-infection in Uganda, hepatitis delta antibodies, hepatitis B virus, HIV

## Abstract

**Background:**

Co-infection with hepatitis B (HBV) and hepatitis D (HDV) is common among human immunodeficiency virus (HIV) infected individuals in developing countries and it aggressively accelerates progression of liver disease to cirrhosis and other complications. There is scarcity of data on HDV in sub-Saharan Africa .We investigated the sero-prevalence and factors associated with HDV antibody among HIV/HBV co-infected patients attending a large urban HIV clinic in Uganda.

**Methods:**

We screened 189 HIV/HBV co-infected individuals for anti-HDV immunoglobulin G (IgG) and performed logistic regression to determine the associated factors. Socio-demographic, clinical data, immunological status, and liver fibrosis (as determined by the Aspartate transaminase to platelet ratio index and transient elastography) were included.

**Results:**

Participants were predominately young and of sound immunologic status (median age 40 years, median CD4 440 cells/µl). 98% were on ART regimens containing anti-HBV active medications (95.2% were on TDF/3TC while 4.8% on 3TC containing regimen). Median duration on ART was 36 months (IQR 22–72). Anti-HDV was detected in 6/198, 3.2% (95% CI 1.14–6.92%), associated with male gender and a duration of more than 5 years since HIV diagnosis.

**Conclusions:**

The sero-prevalence of HDV antibodies among the HIV/HBV co-infected patients is low in a Ugandan urban cohort.

## Introduction

Hepatitis delta virus (HDV) is a defective virus and depends on the hepatitis B surface antigen (HBsAg) for its existence^1^–^3^. Worldwide approximately 15–20 million people have been exposed to HDV infection, which approximately represents 5% of the population of chronic hepatitis B.. Hepatitis D virus infection results in the most aggressive form of chronic viral hepatitis especially in HIV infected persons^4^,^5^. Triple infection with HDV/HBV and human immune deficiency virus (HIV) is common due to shared modes of transmission; mainly through unprotected sexual intercourse and exposure to contaminated blood products^6^,^7^. Current treatment options for HIV/HBV have a limited effect on HDV infection therefore not be adequate if there is HDV coinfection as response to HDV will not be achieved and liver disease progression continues to occur^8^–^12^. There is scarcity of data on HDV in Uganda. To the best of our knowledge there has only been one study which reported HDV antibody prevalence of 30.6 % in the HBsAg positive population and 3.1 % in the general population in the Northern part of Uganda^13^. The aim of this study was to measure the prevalence and to ascertain the factors associated with hepatitis D antibodies among HIV/HBV co-infected adults individuals in Uganda.

## Methods

This cross sectional study was performed at the Infectious Diseases Institute clinic (IDI), Makerere University, Uganda located within Mulago Hospital complex. Ever since it was opened in 2002, this clinic has registered over 10,000 individuals of whom 8,300 are active and are receiving ART. Over the last two years, routine screening of HIV-infected clients for hepatitis B virus has been ongoing and there 250 HIV/HBV co-infected patients attending the IDI clinic.

Between September 2015 and February 2016, we were able to recruit 189 participants. Those that provided written informed consent had their CD4 T-cell count, clinical history relevant to HIV and HBV diagnosis and medication use focusing on ART abstracted from the clinic records. This information was supplemented by a data collection tool that captured socio demographic characteristics and risk factors of HDV transmission. Participants had a physical examination focusing on liver disease and a complete blood count and liver enzyme assays done. In addition, all the participants underwent liver fibrosis assessment using non-invasive approaches; the Aspartate transaminase to platelet ratio index (APRI) and transient elastography with the aim of comparing liver disease severity between the HDV and non HDV infected. We defined liver fibrosis as an APRI score of ≥ 1.5 and transient elastography score of ≥ 9 kpa. Serum was tested for HDV IgG antibodies using the HDV IgG ELISA assay kit (AccuDiag™ HDV-IgG ELISA, Diagnostic automation, inc. Woodland Hills, CA 91367, USA). The manufacturer reports 100% sensitivity and specificity for detecting HDV IgG antibodies using this test.

The study obtained ethical approval from Department of Medicine, School of Medicine Research and Ethics Committee (SOMREC), Makerere University, College of Health Sciences and the IDI scientific review committee.

## Statistics

Data collected was analyzed using STATA software package version 11. Logistic regression was performed to determine the factors associated with anti-HDV. A P-value of < 0.05 was considered to be significant.

## Results

A convenient sample of 189 HIV/HBV co-infected patients was recruited. The study population was composed mainly of young individuals; median age of 40 (IQR 33–46) years and of sound immunological status (median CD4 440 (IQR 155–590 cells/yl). Ninety-eight percent were on ART regimens that contained anti-HBV active medication (95.2% were on TDF/3TC while 4.8% on 3TC containing regimen, 2% were not on ART). Median duration on ART was 36 months (IQR 22–72). The majority (56%) had documented HIV infection for more than 5 years and over two-thirds had been diagnosed with HBV co-infection at least two years prior to this study (table 1).

**Table 1:** Baseline characteristics of the HIV/hepatitis B co-infected patients at IDI clinic, Uganda, (N=189).

Characteristic	N=189 (%)
**Gender**	
Female	80 (42.3%)
Male	109 (57.7%)
**Median age (IQR) years**	40(IQR 33–46)
**Age categories**	
20–30	37 (19.7%)
31–45	103 (54.8%)
45+	48 (25.5%)

**ON ART**	
Yes	186(98.4%)
No	3(1.6%)

**ART regimen**	
**TDF/3TC**	**95.2%**
**3TC**	**4.8%**

**Median duration on ART (IQR)months**	**36 (22–72)**

**Median baseline CD4 count (IQR), cells/µl**	155(46–328)
**Median current CD4 count (IQR)**, cells/µl*	440(155–590)

Median current viral load (IQR) , copies/ml)**	20 (20–75)

**Time since HIV diagnosis**	
≤5 years	82(43.8%)
>5 years	105(56.2%)

**Time since HBV diagnosis**	
≤1 years	73(39%)
≥1 years	114(61%)

* Current CD4 results was available for 188 patients

** Current HIV viral load was available for 174 patients

The prevalence of hepatitis delta antibody was 3.2% (95% confidence interval 1.42–6.92%). All the participants that tested positive for hepatitis delta antibodies were male. They had been diagnosed with HIV and started on ART at least five years prior to this study (table 2).

The liver enzyme levels (ALT, AST), and the platelet count did not significantly differ among the individuals with positive or negative for anti-HDV, neither was there a difference in the HIV viral load and CD4 T-cell count among individuals in these categories.

Furthermore, there was no evidence of liver fibrosis among the individuals that tested positive for anti HDV antibody (normal APRI SCORE (< 1), and 5/6 had a transient elastography score of <7 kpa). One of the participants had invalid fibroscan test. Among the HDV negative participants, liver fibrosis was present in 5 % by the APRI score and 20 % by transient elastography score.

## Discussion

Our study demonstrated a prevalence of hepatitis delta antibodies in HIV/HBV co infected patients in an urban HIV clinic in Uganda of 3.2%. These results are comparable to some other studies in sub-Saharan Africa (SSA) done in similar settings^14^–^16^ but quite different in other selected sub-populations including those with chronic liver disease in West Africa where a high anti-HDV prevalence ranging from 12.2 to 81.7% was documented^17^. Also other studies in Africa have showed varying prevalence rates of HDV ranging from 0–50% in different countries^18^–^20^. Similarly in the developed countries, high anti-HDV prevalence's of 10–15% have been documented in recent studies^1^,^21^,^22^. Reasons for the variations in the prevalence of HDV exposure or infection are not clear. This could be a result of modes of transmission whereby in some of these countries there could be higher rates of IVD use^21^,^22^.

In addition, we have confirmed earlier findings that male gender was significantly associated with anti HDV antibody positivity^23^,^24^.

Within the limits of small numbers in our study, we observed that living with HIV for five or more years was also significantly associated with HDV antibodies despite anti retro viral (ART) use. This data may suggest that ART may not affect HDV antibodies however a larger study is required to assess the impact of ART on HDV infection^8^. However, HIV may increase the risk of HDV acquisition since immunosuppressive states increase the likelihood of chronic hepatitis B which serves as a buffer to hepatitis D acquisition.

This study did not demonstrate significant differences in the liver fibrosis scores among the HDV negative and positive patients, which is in agreement with the clinical and laboratory data that did not suggest that individuals with detectable anti-HDV had a chronic liver disease. However, it may also be possible that the results reflect false positive test for HDV since no confirmation was made using HDV viral loads. On the other had it could also be a result of resolved infection. These have been demonstrated in others studies as well in African settings^14^.

## Limitations

Our study has some limitations. We used a convenient sample of HIV/HBV co-infected individuals that we could access. The few numbers of anti HDV antibody positive individuals identified in this study limited our ability to ascertain the factors associated with triple infection. However, given that similar findings had been recorded elsewhere in East Africa, our findings are likely to be valid. Furthermore, we were unable to perform HDV RNA to confirm current hepatitis delta infection and hence this study was based on HDV exposure and not necessarily current infection.

## Conclusion

The sero prevalence of HDV antibodies among the HIV/HBV co-infected patients is very low in a Ugandan urban cohort as compared with developed countries where intravenous drug use is common. Routine screening for anti-HDV among individuals with HIV/HBV co-infection in this setting may not be cost effective, however in absence of a known etiology, targeted testing can be done for patients with liver disease if not improving or developing liver disease while on a tenofovir based-ART regimen.

Following this pilot, a larger study in which HDV-RNA testing is performed is recommended to assess with better accuracy the magnitude of HDV infection in Uganda.
